# Performance of EQ5D-5L and AHPEQS for measuring outcomes and experiences of patients with peripherally inserted central catheters: a secondary analysis

**DOI:** 10.1007/s11136-026-04264-2

**Published:** 2026-05-03

**Authors:** Emily N. Larsen, Amanda J. Ullman, Nicole Marsh, Ruth Royle, Mari Takashima, Deanne August, Penny Comans Inglis, Nicole Gavin, Amanda Corley, Brighid Scanlon, Doreen Tapsall, Raymond J. Chan, Claire M. Rickard, Joshua Byrnes

**Affiliations:** 1 School of Nursing and Midwifery; School of Medicine and Dentistry; Alliance for Vascular Access Teaching and Research, Griffith University, Brisbane, QLD Australia; 2 Nursing and Midwifery Research Centre; Cancer Care Services, Royal Brisbane and Women’s Hospital, Brisbane, QLD Australia; 3 School of Nursing, Midwifery and Social Work; UQ Centre for Clinical Research, The University of Queensland, Brisbane, QLD Australia; 4 Queensland Children’s Hospital, Children’s Health Queensland, Brisbane, QLD Australia; 5 Faculty of Health; School of Nursing, Queensland University of Technology, Brisbane, QLD Australia; 6 Division of Cancer Services, Princess Alexandra Hospital, Brisbane, QLD Australia; 7 Cancer Futures Institute, Flinders University, Adelaide, SA Australia; 8 Herston Infectious Diseases Institute, Metro North Health, Brisbane, QLD Australia

**Keywords:** EQ5D-5L, AHPEQs, Central venous, Secondary analysis, Patient reported outcome measures

## Abstract

**Purpose:**

Peripherally Inserted Central Catheter (PICC)-associated complications, such as infections and thrombosis, impact significantly upon patients’ experiences and outcomes of care. The purpose of this study was to assess the utility of a patient-reported outcome measure (EuroQol Five Dimension Five Level (EQ5D-5L)), and patient-reported experience measure (Australian Hospital Patient Experience Question Set (AHPEQS)), to discriminate incidence of PICC failure among adults.

**Methods:**

A secondary analysis was undertaken of two large randomised controlled trials, conducted in two adult tertiary hospitals in Queensland, Australia. The EQ5D-5L and AHPEQS instruments were assessed against incidence of three clinical outcomes likely to impact upon self-reported outcomes and experiences: Central Line Associated Bloodstream Infection (CLABSI), venous thrombosis, and all-cause PICC complication. Partial proportional odds, multinomial logistic, linear regression and generalised linear regression models were used to assess instrument performance.

**Results:**

Overall, 984 participants provided baseline EQ5D-5L responses. Subsequently, n=628 completed an EQ5D-5L at study end; n=552 further completed the AHPEQS. EQ5D-5L demonstrated poor discrimination of individual complications (CLABSI, thrombosis), however, there was a significant association (*p*=<0.05) between all-cause PICC complication and: utility (− 0.1, Confidence Interval (CI) − 0.1 to − 0.1); disutility (0.4, CI 0.2–0.7); and self-reported health (– 4.3, CI − 7.8 to − 0.9). AHPEQS demonstrated poor discrimination; one item (‘both physical and emotional harm’) was significantly associated with all-cause PICC complication (1.0, 95%CI 0.1–1.9); CLABSI (2.4, 95%CI 0.7–4.2); and thrombosis (2.0, 95%CI 0.7–3.4).

**Conclusion:**

EQ5D-5L demonstrated moderate suitability for use among patients with PICCs; AHPEQS demonstrated little reliability.

**Supplementary Information:**

The online version contains supplementary material available at 10.1007/s11136-026-04264-2.

## Plain language summary

Patients needing long-term or ‘irritant’ intravenous treatments (like chemotherapy, or antibiotics) often need Peripherally Inserted Central Catheters. One in every four of these devices fail (e., they fall out, or become blocked or infected). As a result, patients can experience both negative physical (discomfort, treatment delay) and psychological (distress) effects. It is important that researchers, doctors, and nurses understand these experiences, to identify where and how care can improve. To do this, special questionnaires called ‘patient-reported outcome measures’ and ‘patient-reported experience measures’ are used to assess health outcomes of patients’ care, and how patients experience this care. These questionnaires can either be very general or relate specifically to a condition (cancer) or intervention (surgery). To date there has been no ‘gold standard’ questionnaire for Peripherally Inserted Central Catheters. Two recent large trials have used general questionnaires as part of their regular data collection. This data was combined and analysed to find out if they were good at picking up whether a patient had experienced Peripherally Inserted Central Catheter complications (i.e., do they do what they are supposed to?). Neither questionnaire was considered ‘gold standard,’ however a few select questions within them appeared useful.

**Supplementary Information:**

The online version contains supplementary material available at 10.1007/s11136-026-04264-2.

## Background

Generic Patient Reported Outcome Measures (PROMs), including multi-attribute utility instruments (MAUI), such as the EuroQol Five Dimension (EQ5D) survey and SF (Short Form) Health Survey, are popular for comparing health states of healthcare consumers experiencing various health conditions (e.g., cancers) [1, 2] and interventions (e.g., orthopaedic surgery) [3, 4]. In practice, they are frequently used for symptom assessment, quality improvement initiatives, and economic evaluations [5]. However, their utility varies greatly, with some health states requiring condition-specific measures (including adaptations of generic PROMs) to ensure the most appropriate and relevant health outcomes are evaluated [6, 7]. An example of this is the European Organisation for Research and Treatment of Cancer Quality of Life Questionnaire (Core-30) (EORTC QLQ C-30). Developed in 1995, this PROM garnered international use; consequently, several systematic reviews and meta-analyses have compared and synthesised the outcomes of this tool, demonstrating that it can enable meaningful judgements upon interventions to improve quality of life [8]. Comparisons between generic and condition-specific tools continue to be made, with condition-specific tools often prevailing in sensitivity within specific disease processes but lacking in their ability to distinguish overall patient health [9].

Patient Reported Experience Measures (PREMs), in contrast, are less frequently reported in published studies, but are nevertheless an important medium on which to judge healthcare quality [10]. PREMs enable consumer self-reporting of interactions with healthcare provision (rather than health status) [11]. Regrettably, the quality of these tools has historically been poor, with a recent systematic review of 88 PREMs identifying that no PREM met all criteria for validity and reliability (assessed against the gold standard ‘COSMIN’ checklist [12]) [11]. Moreover, as fewer studies include PREMs [13], the ability to interpret collated findings, and guide healthcare innovation and quality improvement, is limited.

The insertion and maintenance of Central Venous Access Devices (CVADs), such as Peripherally Inserted Central Catheters (PICCs), is an area where a wide variety of generic and condition-specific PROMs have been used [14]. These include, but are not limited to: SF-12 [15] and SF-36 [16], EORTC QLQ-C30 [17–20] and EORTC QLQ-C15-PAL [21]; and the EQ5D-3L [20]. CVADs are of particular interest for clinicians and researchers seeking to improve quality of care, resulting from their high utility (59% [adult cancer] [22] to 80% [Intensive care Unit] [23]), and commonality of complications (13.4–47% [PICCs]; 13.4–32% [tunnelled CVADs] [20, 24, 25].

A recent systematic review highlighted the broad range of validated and investigator-developed questionnaires, designed to elicit responses related to outcomes and experience of CVADs [14]. However, the use of the term “condition-specific” relates to the primary *underlying* condition of the healthcare consumer (e.g., cancer) rather than the *intervention* of interest (i.e., CVADs). Thus, only two tools were identified to be condition-related *to CVADs*: the “Questionnaire for Acceptance of and Satisfaction with Implanted Central Venous Catheter” (QASSICC); and the “Quality of Life Assessment, Venous Device - Port" (QLAVD-P). Neither tool met all the criteria for validity and reliability.

Given the apparent need for innovation and quality improvement in this area, a reliable and consistently applied PROM and PREM is essential to guide future healthcare delivery. The utility of generic versus condition-specific tools, however, is yet to be determined. Existing tools such as the EQ5D-Five Level (5L) and the “Australian Hospital Patient Experience Question Set” (AHPEQS) [26] are widely used in Australian healthcare settings and present as a reasonable option for generic tools to be applied to this area, capitalising on existing systems to reduce data collection burden. Thus, the aim of this study was to assess the utility of a generic PROM (EQ5D-5L) [27] and PREM (AHPEQS) for adult patients with PICCs.

## Methods

### Data collation

Data from two multi-site randomised controlled trials (RCTs) comparing (1) PICC dressings and securements (ACTRN12616000315415) [28], and (2) PICC catheter materials (ACTRN12619000022167) [29] were collated for this secondary analysis. The PICC dressing and securement RCT, a two-by-two factorial trial, compared the use of integrated securement devices and standard polyurethane dressings with sutureless securement devices (with and without chlorhexidine impregnated disc dressings); PICCs were comprised of standard polyurethane or hydrophobic materials [28]. The PICC materials RCT compared the use of standard polyurethane, hydrophobic polyurethane, and chlorhexidine-coated polyurethane PICCs [29].

The studies were conducted between June 2016 and September 2020 [28]; and September 2019 and December 2022 [29] for the dressing and PICC material trials, respectively. Both RCTs included patients with PICCs, receiving either inpatient or outpatient care from two quaternary adult hospitals (~ 1000 beds), and one tertiary paediatric hospital (~ 350 beds) in Queensland, Australia. Study participants were drawn from a large breadth of disciplines, including cancer care, medical, surgical, and intensive care (including trauma). Participants frequently transitioned between inpatient and outpatient care and were followed for a period of six and eight weeks for the dressing and PICC material trials, respectively (or until PICC removal, whichever occurred first) [28, 29]. Data for participant demographics, device details and patient outcomes were from a variety of sources, including patient self-reporting, clinician reporting, medical notes, and pathology results.

Paediatric data (< 17 years), including results of EQ-5D *Youth*, were excluded for the purposes of this secondary analysis. As this was a secondary analysis of existing data, a sample size was not calculated; rather, all participants who had provided responses in the original studies were included.

Eligible patients were recruited prior to PICC insertion [28, 29] or within 24 h of insertion [28]. All data was initially entered on REDCap electronic data capture [30]. Demographic (e.g., gender, age) and device data (e.g., date/time of PICC insertion, number of insertion attempts) were collated if collected homogenously between the two included RCTs. Regular checks and standardised outcome data (e.g., reason for PICC removal, local and systemic complications) were collected prospectively [28, 29].

### Instruments

Throughout, each *question* within the respective instruments will be termed ‘items,’ and the values recorded from responses on item scales will be termed ‘scores.’

### EQ5D-5L

EQ5D-5L is a MAUI that collects self-reported responses of healthcare consumers’ mobility, self-care, usual activities, pain/discomfort, and anxiety/depression (five domains), across five levels ranging from “no problems” to “extreme problems.” [27] The EQ5D-5L also includes a single visual analogue scale (VAS) for a self-reported health status (0 - 100; worst to best health). The responses can be scored using a preference-based algorithm to produce a continuous ‘summary index value’ (utility value). Using the Australian EQ5D-5L algorithm, the EQ5D-5L may account for up to 243 different health states (1.0, perfect health to − 0.217, worse than death) [31]. The tool is available in four varieties: three-level (adult >16 years, EQ5D-3L), five-level (adult >16 years, EQ5D-5L), youth three-level (≤16 years, EQ-5D-Y-3L), and youth five-level (≤16 years, EQ-5D-Y-5L) [27]; translated into over 150 languages.

The EQ5D-5L was selected by investigator consensus for the dressing and securement RCT [28]; and in the following PICC material RCT [29] after anecdotal feedback of patient acceptability, and investigative team feedback. The impacts of CVADS (and their associated complications) upon self-reported outcomes of four of the five EQ5D-5L domains had been well established; in particular, its impact upon activities (and instrumental activities-) or daily living (related domain/s: self-care; usual activities); pain/discomfort; and psychological burden (related domain: anxiety/depression) [32]. Notably, the EQ5D-3L had previously been disseminated and collected within a large multi-centre RCT, which compared the use of three CVADs (PICCs, tunnelled cuffed catheters, and implanted ports) for the delivery of cancer therapies [20]. The EQ5D-5L had also been tested for use within the context of Peripheral Intravenous Catheters, with limited ability to discriminate differences in experiences and outcomes of those experiencing multiple insertion attempts or device failure, or detect changes over time [33].

### AHPEQS

The AHPEQS, released by the Australian Commission on Safety and Quality in Health Care, was developed between 2015 and 2017 [26]. This 12-item (five-level) questionnaire comprises of items phrased as statements (from the patient’s perspective), including items such as “*I received pain relief that met my needs* “and *“When I was in hospital I felt confident in the safety of my treatment and care*” [26]. A scoring algorithm was not available/used for this tool.

The AHPEQS was selected by investigator consensus for the PICC material RCT 29, following a decision to ensure adequate representation of patient reported *experiences*, as well as outcomes. The items included in AHPEQS, particularly those items aligned with staff interactions and quality of care (e.g., “*My views and concerns were listened to” and “When I was in the hospital*,* I felt confident in the safety of my treatment and care”)* aligned with documented self-reported experience of patients with CVADs (with and without complications). For example, several qualitative studies have found that patients had previously reported “*mistrust*” and that “*staff at times lacked experience*” [32]. The AHPEQS was similarly previously tested for discrimination within the context of Peripheral Intravenous Catheters, demonstrating some positive results, e.g., patients experiencing device failure were more likely to report “*unexpected physical or emotional harm*.” [33].

## Survey collection

Methods of PROM (EQ5D-5L) and PREM (AHPEQS) questionnaire data collection were as follows: all recruited participants (n=1098) within the PICC material RCT (where appropriate) were approached to provide responses to the EQ5D-5L at (i) PICC insertion (baseline) and (ii) removal (reliant upon participant availability) [29]; and AHPEQs questionnaires at removal only (reliant upon participant availability) [34]. In contrast, a convenience sample of n=70 from the dressing and securement RCT were approached to complete the EQ5D-5L only [no AHPEQs tool] at study (i) enrolment and (ii) completion (reliant upon participant availability) [28]. Study completion occurred for both studies at: the time of device failure; the time of completion of therapy (and device removal); or at a maximum follow-up period of eight weeks [28, 29].

As this was a secondary outcome for each of the corresponding RCTs, no additional data were collected regarding the reason for (1) T0 EQ5D-5L non-completion, or (2) (T1) EQ5D-5L or AHPEQS non-completion. Reasons were anecdotally reported to have included refusal, time constraints, and transfer to another facility.

### Outcome of interest

The performance of the EQ5D-5L and AHPEQS instruments were considered for three outcomes of interest:

1. Central Line Associated Bloodstream Infection (CLABSI) (yes, no), defined according to the National Healthcare and Safety Network (NHSN). Standard criteria required a blood culture positive for either (1) a recognised pathogen (single specimen) or (2) two common commensal organisms grown from two samples, from a symptomatic patient, within two calendar days of one another; in the absence of another source of infection [35].
2. Confirmed thrombosis (yes, no), determined by ultrasound or other imaging, occurring at any time during the PICC dwell. These were either (1) clinically symptomatic and classified by a radiologist [34]; or (2) classified by a suitably qualified medical officer (with or without the presence of clinical symptoms) [28].
3. All-cause PICC complication (yes, no): this was a composite measure of all modes of PICC complications resulting in PICC dysfunction with or without the requirement for PICC removal. This included: CLABSI [35], local infection (NHSN criteria) [35], dislodgement (change in PICC length, including complete accidental removal) (dressing RCT only) [28], occlusion (complete or partial), fracture (dressing RCT only) [28], and confirmed thrombosis.

### Data analysis

Analyses were performed using Stata statistical software (v16.1, StataCorp). First, EQ5D-5L utility scores were computed [31], with disutility scores (1.0 minus utility score) analysed with generalised linear regression models (EQ5D-5L). Partial proportional odds models were used for ordered polytomous EQ5D-5L and AHPEQS items; multinomial logit models were used for unordered polytomous items. Linear models were used for EQ5D-5L utility scores and visual analogue scores; generalised linear regression models were used for disutility scores. In each analysis, the dependent variable was a dimension of an instrument or an overall score. For EQ5D-5L, the follow-up time point was used. Covariables included: the instrument dimension measured at baseline (EQ5D-5L only); age; gender; hospital; inpatient or outpatient status (at PICC insertion); whether the participant had four or more comorbidities (at PICC insertion); whether insertion required multiple (>1) attempts; radiologist inserter; side of device insertion (right/left); device dwell time (in days); device brand; PICC complications (CLABSI; local infection; dislodgement; occlusion; fracture; thrombosis). Stepwise regression was used to build each regression model from the list of candidate variables. A significance level of* p* < 0.10 was used for entry and removal of covariables from the models. The parallel lines assumption was tested using a 0.05 level of significance. In all final models,* p* < 0.05 was considered statistically significant. A sensitivity analysis was conducted to assess differences in demographics and outcomes of interest between respondents who did and did not complete a follow-up EQ5D-5L (T1). No missing data was imputed. 

Responsiveness statistics for utility were also computed. The formula for these statistics was as follows: Effect size (ES) = D/SD; and Standardised response mean (SRM) = D/SD*, where D = mean score change, SD = standard deviation of the baseline score, and SD* = standard deviation of the score change. The ES was considered ‘trivial’ if < 0.2; ‘small’ if ≥ 0.2 and < 0.5; moderate if “≥0.5 and < 0.8; and large if ≥ 0.8 [36].

## Results

Of 1723 adults enrolled across both trials, a total of 984 (57.1%) adult participants completed the baseline EQ5D-5L (T0) at, or close to, the time of PICC insertion (Table 1). Following this, 628 and 552 participants completed the follow-up EQ5D-5L and AHPEQS instruments, respectively (EQ5D-5L T1 attrition, n=356; 36.2%). Most participants were male (61%; baseline cohort), with a mean age of 57 (Standard Deviation (SD), 15). PICC insertion commonly occurred while the participant was an inpatient (67%, at baseline); with 46% of participants admitted for an oncological or haematological condition. The average dwell time was 29 days (range 1–73 days), and all-cause complications occurred in 23.4% of the baseline population (24.0%, EQ5D-5L T1 cohort; 23.7% AHPEQS cohort). The most frequently occurring individual complication was occlusion (7.7%, 9.1%, 9.8% for EQ5D-5L T0, EQ5D-5L T1 and AHPEQS, respectively). CLABSI occurred for n=20 (2%) participants of the baseline population (1.9% EQ5D-5L T1 cohort; 1.5% AHPEQS cohort); and thrombosis occurred among n=42 (4.3%) of the baseline population (3.3% for both EQ5D-5L T1 and AHPEQS cohorts).

**Table 1:** Participant and device characteristics

Participant characteristics	EQ5D-5L T0 (*N* = 984)	EQ5D-5L T1 (*N* = 628)	AHPEQS (*N* = 552)
	*n* (%)*	*n* (%)*	*n* (%)*
Gender (male)	598 (60.8)	380 (60.5)	345 (62.5)
Age in years (mean, SD) Range	57, 15 17–98	58, 15 17–92	59, 15 17–85
*Hospital*			
PAH	580 (58.9)	373 (59.4)	356 (64.5)
RBWH	404 (41.0)	255 (40.6)	196 (35.5)
*Setting (at PICC insertion)*			
Inpatient	659 (67.0)	423 (67.4)	378 (68.5)
Outpatient	325 (33.0)	205 (32.6)	174 (31.5)
*Reason for admission*			
Surgical	269 (27.3)	175 (27.9)	165 (29.9)
Oncology	255 (25.9)	159 (25.3)	142 (25.7)
Haematology	197 (20.0)	127 (20.2)	82 (14.9)
Medical	58 (5.9)	34 (5.4)	34 (6.2)
Gastroenterology	59 (6.0)	39 (6.2)	39 (7.1)
Cardiac	40 (4.1)	26 (4.1)	25 (4.5)
Respiratory	19 (1.9)	14 (2.2)	13 (2.4)
Other	87 (8.8)	54 (8.6)	52 (9.4)
Multiple insertion attempts (yes)	143 (14.5)	81 (12.9)	64 (11.6)
Three or more insertion attempts	59 (6.0)	35 (5.6)	28 (5.1)
*Number of attempts*			
1	835 (84.9)	546 (86.9)	487 (88.2)
2	84 (8.5)	46 (7.3)	36 (6.5)
3	48 (4.9)	31 (4.9)	24 (4.4)
4	8 (0.8)	2 (0.3)	2 (0.4)
5	3 (0.3)	2 (0.3)	2 (0.4)
Unknown	6 (0.6)	1 (0.2)	1 (0.2)
Current infection (yes)	281 (28.6)	175 (27.9)	157 (28.4)
*Comorbidities*			
1	167 (17.0)	93 (14.8)	75 (13.6)
2	146 (14.8)	89 (14.2)	85 (15.4)
3	75 (7.6)	50 (8.0)	44 (8.0)
>3	488 (49.6)	322 (51.3)	306 (55.4)
None	108 (11.0)	74 (11.8)	42 (7.6)
*PICC materials*			
Polyurethane	311 (31.6)	195 (31.0)	188 (34.1)
Hydrophobic	370 (37.6)	248 (39.5)	184 (33.3)
Chlorhexidine	300 (30.5)	185 (29.5)	180 (32.6)
Unknown	3 (0.3)	0 (0.0)	0 (0.0)
*Side of placement*			
Left	643 (65.4)	409 (65.1)	364 (64.9)
Right	338 (34.4)	219 (34.9)	188 (34.1)
Unknown	3 (0.3)	0 (0.0)	0 (0.0)
*Number of lumens*			
1	15 (1.5)	8 (1.3)	8 (1.5)
2	966 (98.2)	620 (98.7)	544 (98.6)
Unknown	3 (0.3)	0 (0.0)	0 (0.0)
Ease of insertion^ǂ^ (mean, SD)	85.9, 22.6	87.1, 22.1	87.3, 21.6
Inserter			
Radiographer	769 (78.2)	492 (78.3)	454 (82.3)
Nurse	205 (20.9)	132 (21.0)	98 (17.8)
Doctor	5 (0.5)	3 (0.5)	0 (0.0)
Other	1 (0.1)	1 (0.2)	0 (0.0)
Unknown	4 (0.4)	0 (0.0)	0 (0.0)
*Intervention/control#*			
*PICNIC*	*n* = 915	*n* = 568	*n* = 552
Polyurethane PICC	308 (33.7)	189 (33.2)	183 (33.2)
Hydrophobic PICC	302 (33.0)	190 (33.5)	185 (33.5)
Chlorhexidine PICC	305 (33.3)	189 (33.3)	184 (33.3)
*PISCES*	*n* = 69	*n* = 60	*n* = 0
ISD (+ Chlorhexidine impregnated patch)	20 (29.0)	19 (31.7)	0 (0.0)
ISD (no Chlorhexidine impregnated patch)	20 (29.0)	15 (25.0)	0 (0.0)
SSD (+ Chlorhexidine impregnated patch)	14 (20.3)	13 (21.7)	0 (0.0)
SSD (no Chlorhexidine impregnated patch)	15 (21.7)	13 (21.7)	0 (0.0)

Device characteristics	n (%)*	n (%)*	n (%)*
*Outcomes*			
Dwell time (in days) (mean, SD)	29.0, 18.7	29.6, 18.8	28.6, 18.5
All-cause PICC complication	230 (23.4)	151 (24.0)	131 (23.7)
*Complications^:*			
CLABSI	20 (2.0)	12 (1.9)	8 (1.5)
Local	4 (0.4)	3 (0.5)	3 (0.5)
Occlusion	76 (7.7)	57 (9.1)	54 (9.8)
Dislodgement	30 (3.1)	21 (3.3)	19 (3.4)
Confirmed thrombosis	42 (4.3)	21 (3.3)	18 (3.3)
Fracture~	4 (0.4)	1 (0.2)	0 (0.0)
Skin reaction (yes)~	14 (1.4)	13 (2.1)	0 (0.0)

*Unless otherwise noted; ^more than one category possible; ǂscale 0–100, higher score indicates greater ease, number of observations 907, 566 and 550 for EQ5D-5L T0, EQ5D-5L T1 and AHPEQS, respectively; #study group/arm to which patient allocated; ~dressing RCT only, number of observations 69, 60 and 0 for EQ5D-5L T0, EQ5D-5L T1 and AHPEQS, respectively; EQ5D-5L = EuroQol Five Dimension, Five Level; T0 = baseline timepoint; T1 = follow-up timepoint; AHPEQS = Australian Hospital Patient Experience Question Set; CLABSI = central line-associated bloodstream infection; ISD = integrated securement dressing; SSD = polyurethane dressing with a sutureless securement device; PICC = peripherally inserted central catheter; SD = standard deviation

The mean EQ5D-5L VAS improved from 62.3 (SD 20.3) at baseline, to 68.8 (SD 19.7) at follow-up (EQ5D-5L T1). Similarly, across all EQ5D-5L domains, responses at T1 had improved with 8% (12.4% T0), 4.5% (9.3% T0), 15.8% (24.1% T0), 5.3% (9.8% T0), 1.3% (3% T0) participants reporting ‘severe’ or ‘extreme’ problems, for mobility, personal care, usual activities, pain/discomfort, and anxiety/depression, respectively (Table 2). Consequently, the overall EQ5D-5L utility score improved from 0.75 (SD 0.28), to 0.83 (SD, 0.23) at T0 and T1, respectively.

Upon sensitivity analysis, EQ5D-5L T1 non-respondents (attrition; n=356) demonstrated similar baseline demographics and device details to EQ5D-5L T1 respondents (Supplementary Table 1). Some differences were noted in age (T1 respondents: mean age, 58 (SD15); T1 non-respondents, 55 (SD16) (*p*=0.002); occurrence of multiple insertion attempts (12.9% T1 respondents v. 17.4% T1 non-respondents;* p*=0.04); and incidence of skin reactions (21.7% T1 respondents v. 11.1% T1 non-respondents;* p*= 0.001). T0 EQ5D responses were also similar between respondents and non-respondents for all domains except Usual Activities (*p*= 0.07).

Individual AHPEQS items demonstrated a ceiling effect, with over 80% of participants reporting that their ‘views and concerns were *always* listened to’ (81.9%, *n* = 452); ‘individual needs were *always* met’ (81.5%, *n* = 450); ‘*always* felt cared for’ (85.7%, *n* = 473); ‘*always* received pain relief that met (their) needs’ (81%, n = 447); ‘*always* felt confident in the safety of (their) treatment and care’ (87.1%, n = 481); and reporting *no ‘*experiences of unexpected harm of distress as a results of (their) treatment or care’ (89.5%, n = 494).

**Table 2:** Responses: EQ5D-5L, AHPEQS

Instrument	Scale (*n* (%))					
EQ5D	No	Slight	Moderate	Severe	Extreme	Score (mean, SD)
T0 (*N* = 984)						
Mobility	560 (56.9)	195 (19.8)	107 (10.9)	50 (5.1)	72 (7.3)	
Personal care	579 (58.8)	200 (20.3)	113 (11.5)	52 (5.3)	40 (4.1)	
Usual activities	281 (28.6)	211 (21.4)	255 (25.9)	86 (8.7)	151 (15.4)	
Pain/discomfort	309 (31.4)	315 (32.0)	264 (26.8)	90 (9.1)	6 (0.6)	
Anxiety/ depression	376 (38.2)	412 (41.9)	166 (16.9)	24 (2.4)	6 (0.6)	
Health today (VAS)						62.3, 20.3
Utility score						0.75, 0.28
Disutility score						0.25, 0.28
T1 (*N* = 628)						
Mobility	397 (63.2)	108 (17.2)	73 (11.6)	30 (4.8)	20 (3.2)	
Personal care	406 (64.7)	129 (20.5)	65 (10.4)	17 (2.7)	11 (1.8)	
Usual activities	192 (30.6)	188 (29.9)	149 (23.7)	41 (6.5)	58 (9.2)	
Pain/discomfort	259 (41.2)	230 (36.6)	106 (16.9)	28 (4.5)	5 (0.8)	
Anxiety/ depression	353 (56.2)	207 (33.0)	60 (9.6)	4 (0.6)	4 (0.6)	
Health today (VAS)						68.8, 19.7
Utility score						0.83, 0.23
Disutility score						0.17, 0.23

AHPEQS (*N* = 552)*	Always	Mostly	Sometimes	Rarely	Never	Didn’t apply
Views and concerns	452 (81.9)	85 (15.4)	8 (1.5)	3 (0.5)	2 (0.4)	0 (0.0)
Individual needs	450 (81.5)	84 (15.2)	12 (2.2)	1 (0.2)	0 (0.0)	.
Staff explanation (*n* = 13)	1 (7.7)	2 (15.4)	7 (53.9)	3 (23.1)	0 (0.0)	.
Cared for	473 (85.7)	67 (12.1)	7 (1.3)	2 (0.4)	0 (0.0)	.
Decision making	438 (79.4)	85 (15.4)	19 (3.4)	8 (1.5)	1 (0.2)	.
Informed	432 (78.3)	95 (17.2)	18 (3.3)	5 (0.9)	1 (0.2)	.
Inter-staff communication	437 (79.2)	82 (14.9)	23 (4.2)	5 (0.9)	1 (0.2)	1 (0.2)
Pain relief	447 (81.0)	42 (7.6)	16 (2.9)	3 (0.5)	4 (0.7)	36 (6.5)
Confidence in safety	481 (87.1)	52 (9.4)	10 (1.8)	3 (0.5)	0 (0.0)	.

	Physical harm	Emotional distress	Both	No
Unexpected harm	13 (2.4)	22 (4.0)	19 (3.4)	494 (89.5)

	Yes	No	Not sure	Don’t want to discuss
Harm discussed (*n* = 48)	31 (63.3)	11 (22.5)	5 (10.2)	1 (2.0)

	Very good	Good	Average	Poor	Very poor
Overall quality	348 (63.0)	183 (33.2)	15 (2.7)	3 (0.5)	0 (0.0)

*Not all rows sum to N due to responses missing at random; EQ5D-5L= EuroQol Five Dimension, Five Level; T0 = baseline timepoint; T1 = follow-up timepoint; AHPEQS = Australian Hospital Patient Experience Question Set; VAS = visual analogue scale; ‘.’ indicates cell intentionally blank

Thrombosis (confirmed) and CLABSI were not significantly associated with any EQ5D-5L items or overall utility/disutility scores (Table 3). Participants experiencing all-cause PICC complications, in comparison, demonstrated lower utility scores (− 0.1, 95% Confidence Interval (CI) − 0.1 to − 0.1;* p*=0.001), and higher disutility scores (0.4, 95%CI 0.2–0.7;* p*=0.002). Similarly, self-reported health status was lower among those experiencing all-cause PICC complications (− 4.3, 95%CI − 7.8 to − 0.9;* p*=0.01). The domains for which there was a statistically significant negative impact on participants experiencing all-cause PICC complication, included mobility (0.6, 95%CI 0.2–1.0;* p*=0.002), personal care (0.7, 95%CI 0.3–1.1;* p*=<0.001), and usual activities (0.6, 95%CI 0.2–0.9;* p*=0.001). The ES for EQ5D-5L was categorised as ‘small’ at 0.27; and the Standardised Response Mean was 0.28.

**Table 3:** EQ5D-5L discrimination results

Dimension/score (*N* = 628)	Outcome (95% CI); *p* value		
	All-cause PICC complication	CLABSI	Thrombosis
Dimension			
Mobility	**0.6 (0.2–1.0); 0.002**	0.1 (− 1.0 to 1.2); 0.84	NC
Personal care	**0.7 (0.3–1.1); <0.001**	− 0.2 (− 1.4 to 1.0); 0.76	NC
Usual activities	**0.6 (0.2–0.9); 0.001**	NC	0.4 (− 0.4 to 1.2); 0.32
Pain/discomfort	0.3 (− 0.1–0.7); 0.07	0.5 (− 0.5 to 1.5); 0.35	NC
Anxiety/ depression	0.2 (− 0.1–0.6); 0.25	0.5 (− 0.6 to 1.7); 0.37	0.1 (− 0.8 to 0.9); 0.96
Health today (VAS)	**− 4.3 (− 7.8 to − 0.9); 0.01**	− 5.5 (− 16.8 to 5.7); 0.34	− 6.4 (− 14.9 to 2.2); 0.15
Utility score	**− 0.1 (− 0.1 to − 0.1); 0.001**	− 0.1 (− 0.2 to 0.1); 0.83	− 0.1 (− 0.2 to 0.1); 0.29
Disutility score	**0.4 (0.2–0.7); 0.002**	0.1 (− 0.7 to 0.9); 0.84	0.3 (− 0.3 to 0.9); 0.35

EQ5D-5L, EuroQol Five Dimension; Five Level; CI,confidence interval; CLABSI, central line associated bloodstream infection; VAS, visual analogue scale; NC, not calculable; bold case indicates statistical significance <0.05

A single AHPEQS item (‘experienced unexpected harm or distress as a result of (their) treatment or care’; both physical and emotional harm) was significantly associated with poorer scores across all three outcomes of interest: all-cause PICC complication (1.0, 95%CI 0.1–1.9; *p* = 0.05); CLABSI (2.4, 95%CI 0.7–4.2; *p* = 0.005); and thrombosis (2.0, 95%CI 0.7–3.4; *p* = 0.004). Those experiencing CLABSI also reported statistically lower scores for ‘overall quality of the treatment (they) received’ (1.7, 95%CI 0.3–3.2; *p* = 0.02). Participants experiencing thrombosis further reported negative experiences with “pain relief (meeting) my needs” (1.0, 95%CI 0.1–1.9; *p* = 0.04). Finally, those experiencing all-cause PICC complications were statistically significantly more likely to report *not* being “involved as much as (they) wanted in making decisions about (their) treatment and care” (0.8, 95%CI 0.3–1.2; *p* = 0.001) (Table 4).

**Table 4:** AHPEQS discrimination results

Dimension/score (*N* = 552)	Outcome (95% CI); *p* value		
	All-cause PICC complication	CLABSI	Thrombosis
Views and concerns	NC	0.9 (− 0.5 to 2.4); 0.19	0.9 (− 0.1 to 1.9); 0.08
Individual needs	**0.6 (0.1–1.1); 0.01**	0.9 (− 0.5 to 2.4); 0.19	0.9 (− 0.1 to 1.9); 0.08
Staff explanation (*n* = 13)	− 1.0 (− 3.4 to 1.4); 0.41	NC	− 0.1 (− 3.5 to 3.5); 1.00
Cared for	0.5 (− 0.1 to 1.0); 0.07	NC	1.4 (0.4–2.4); 0.006
Decision making	**0.8 (0.3–1.2); 0.001**	0.7 (− 0.7 to 2.1); 0.32	0.7 (− 0.3 to 1.7); 0.17
Informed	0.3 (− 0.2 to 0.7); 0.29	0.3 (− 1.3 to 1.9); 0.73	0.6 (− 0.4 to 1.6); 0.24
Inter-staff communication	NC	0.5 (-1.1 to 2.1); 0.54	0.8 (− 0.2 to 1.8); 0.12
Pain relief	NC	− 0.4 (− 2.5 to 1.8); 0.74	**1.0 (0.1–1.9); 0.04**
Confidence in safety	0.4 (− 0.2 to 1.0); 0.17	0.2 (− 2.0 to 2.3); 0.87	0.6 (− 0.7 to 1.9); 0.38
Unexpected harm			
Physical vs. No harm	**1.7 (0.6–2.9); 0.003**	− 10.1 (− 868 to 848); 0.98	1.2 (− 0.9 to 3.3); 0.26
Emotional vs. No harm	0.5 (− 0.4 to 1.4); 0.23	1.5 (− 0.7 to 3.7); 0.17	1.4 (− 0.2 to 3.0); 0.08
Both vs. No harm	**1.0 (0.1–1.9); 0.045**	**2.4 (0.7–4.2); 0.005**	**2.0 (0.7–3.4); 0.004**
Harm discussed (*n* = 48)			
No vs. Yes	**− 2.4 (− 4.5 to − 0.2); 0.03**	− 14.7 (− 3532 to 3503); 0.99	− 16.5 (− 6988 to 6955); 0.99
Not sure vs. Yes	0.3 (− 1.6 to 2.3); 0.73	1.3 (− 1.3 to 3.9); 0.34	1.8 (− 0.3 to 4.0); 0.10
Don’t want to vs. Yes	− 14.9 (-3,346 to 3,317); 0.99	− 14.7 (− 11,682 to 11,653); 0.99	− 16.5 (− 23,137 to 23,104); 0.99
Overall quality	0.1 (− 0.2 to 0.5); 0.54	**1.7 (0.3–3.2); 0.02**	0.7 (− 0.3 to 1.6); 0.15

AHPEQS, Australian hospital patient experience question Set; CI, confidence interval; CLABSI, central line associated bloodstream infection; NC, not calculable; bold case indicates statistical significance *≤* 0.05

## Discussion

This secondary analysis evaluated the use of EQ5D-5L and AHPEQS among a large cohort of adults with PICCs, identifying a small selection of items able to discriminate between patients experiencing positive versus negative clinical outcomes. Participants experiencing all-cause PICC complication demonstrated lower EQ5D-5L utility (− 0.1, 95%CI − 0.1 to − 0.1; 0.001), and higher disutility scores (0.4, 95%CI 0.2–0.7; 0.002). This is a clinically important difference; within the range of the widely recognised ‘minimally important difference’ for EQ5D (mean 0.074; range − 0.011 to − 0.140) [36]. In contrast, the EQ5D-5L was unable to discriminate the incidence of individual modes of PICC complication known to impact significantly upon patient experiences and outcomes (CLABSI, thrombosis) [37-39]. However, this finding may be a consequence of low incidence (1.9%, and 3.3% for CLABSI and thrombosis, respectively), rather than tool performance.

Notably, the observed EQ5D-5L scores improved over time, with utility increasing from 0.75 (SD 0.28)–0.83 (SD 0.23). The reasons for this improvement may be multifaceted. CVADs such as PICCs are typically inserted during an acute episode of illness (e.g., infection), following previous CVAD failure, or at commencement of treatment protocols for new diagnoses (e.g., chemotherapy; immunotherapy) when an infusion is required for more than 7 days [40]. Thus, improved EQ5D-5L may reflect patient stabilisation and improved overall health following treatment. This improvement may also reflect improved adaptability to PICC dwell, however as the baseline EQ5D-5L was delivered shortly following PICC insertion it is not possible to fully determine its influence.

The key clinical implication of our findings is that PICC failure meaningfully affects HRQoL. It is therefore reasonable to anticipate that other related factors, such as device appropriateness and selection, lumen number, and insertion site, may also influence HRQoL. However, a large multi-centre RCT (n=1061) comparing three CVADs (PICCs, cuffed tunnelled catheters, and implanted ports) demonstrated no significant differences in HRQoL assessed using the EQ5D-3L [20]. Qualitative data however suggests that CVADs can in fact significantly affect healthcare consumers' lived experiences, influencing daily activities, social interactions, and contributing to psychological burden [32]. This apparent disconnect in findings may result from the use of generic PROMs, like EQ5D, for CVADs, which has largely outstripped the use of condition-specific PROMs in published works [14]. Limited analyses have been conducted to establish their validity and reliability in this context [14]. Moreover, the use of PREMs has been largely overlooked [14]. Considering the high prevalence of CVADs, this presents an important gap. Thus, establishing a gold-standard PROM for patients with CVADs (including PICCs) remains a priority, however the items included in such a PROM and PREM are critical and should be thoughtfully considered.

In our study, only one item within AHPEQS (‘*unexpected harm’*) demonstrated the ability to discriminate participants experiencing all-cause PICC complications, CLABSI, *and* thrombosis. Notably, this same item similarly demonstrated usefulness in a secondary analysis of AHPEQS use in the context of *Peripheral Intravenous Catheters* [33]. Notwithstanding the floor effect for this item (with 90% stating ‘no’ unexpected harm), it is notable that 9.8% (n = 54) reported physical harm (2.4%), emotional distress (4%) or both (3.5%). Given the widespread use of CVADs in clinical practice, these self-reports of harm should be a key focus for quality improvement efforts. As attention shifts to self-reported measures, researchers and tertiary facilities continue to focus upon *outcomes*, which often align with policy priorities and key performance indicators [41], rather than consumer *experiences*. Despite this, both PROMs and PREMs, administered well, can inform healthcare quality improvement, improve individual patient care, demonstrate *value* of healthcare interventions, and positively influence policy [42].

### Limitations

While findings are limited by minor variations in data collection methods, and outcomes of interest between the two included RCTs, the large sample with which tool discrimination and responsiveness could be analysed presented a significant strength to the methods and subsequent findings. For both included RCTs (conducted exclusively in Australia), a suitably trained Research Nurse was responsible for PROM and PREM administration, therefore findings may not be replicated among hospitals in which PROM and PREM collection is dispersed hospital or health services wide. While there was significant attrition between EQ5D-5L T0 (baseline) and T1 (study completion, PICC removal), general homogeneity between patient demographics and rates of outcomes of interest between T0 and T1 suggests the impact of this attrition was minimal. Finally, the impact of various diagnosis, and treatment factors upon HRQoL (for example admitted [inpatient] days) may have been underestimated due to the inherent focus on factors related to the* PICC* specifically.

## Conclusions

Patients experiencing all-cause PICC complications demonstrated lower EQ5D-5L utility scores, indicating moderate usefulness of the instrument in acute clinical settings for this population. However, neither EQ5D-5L nor AHPEQS presented as a reliable (whole) tool from which to understand healthcare consumers' outcomes and experiences; this was particularly true for individual complications. Given the high prevalence of CVADs in modern tertiary care, the high incidence of CVAD failure, and the significant clinical and personal outcomes which may arise from critical modes of failures (such as thrombosis and CLABSI), high priority should be given to the assessment of other existing tools used in this context, and (where necessary) the development of a condition-specific PROM and PREM.

## Supplementary Information

Below is the link to the electronic supplementary material.

Supplementary Material 1

## Data Availability

The datasets and code used for this psychometric analysis are available from the corresponding author on reasonable request, and in accordance with original HREC/s restrictions for use.
