# Supplementary material for: Performance of EQ5D-5L and AHPEQS for measuring outcomes and experiences of patients with peripherally inserted central catheters: a secondary analysis
Source: Qual Life Res. 2026 May 3;35(6):150. doi: 10.1007/s11136-026-04264-2 (PMC13136200; doi:10.1007/s11136-026-04264-2)
Supplement: Supplementary file 1 — Supplementary Material 1 [file 11136_2026_4264_MOESM1_ESM.docx]

**Supplementary Table 1** Comparison of the baseline characteristics and clinical outcomes of EQ5D-5L responders vs non-responders at follow-up

|  | **EQ5D-5L RESPONDERS**  **(N = 628)** | **EQ5D-5L NON-RESPONDERS**  **(N = 356)** |  |
| --- | --- | --- | --- |
| **PARTICIPANT BASELINE CHARACTERISTICS** | **n (%)*** | **n (%)*** | ***p*-value**^†^ |
| Gender (male) | 380 (60.5) | 218 (61.2) | 0.92 |
| Age in years (mean, SD)  *Range* | 58, 15  *17 to 92* | 55, 16  *18 to 98* | 0.002 |
| Hospital |  |  | 0.46 |
| PAH | 373 (59.4) | 207 (58.2) |  |
| RBWH | 255 (40.6) | 149 (41.9) |  |
| Setting |  |  | 0.58 |
| Inpatient | 423 (67.4) | 236 (66.3) |  |
| Outpatient | 205 (32.6) | 120 (33.7) |  |
| Reason for admission |  |  | 0.99 |
| Surgical | 175 (27.9) | 94 (26.4) |  |
| Oncology | 159 (25.3) | 96 (27.0) |  |
| Haematology | 127 (20.2) | 70 (19.7) |  |
| Medical | 34 (5.4) | 24 (6.7) |  |
| Gastroenterology | 39 (6.2) | 20 (5.6) |  |
| Cardiac | 26 (4.1) | 14 (3.9) |  |
| Respiratory | 14 (2.2) | 5 (1.4) |  |
| Other | 54 (8.6) | 33 (9.3) |  |
| Multiple insertion attempts (yes) | 81 (12.9) | 62 (17.4) | 0.04 |
| Three or more insertion attempts | 35 (5.6) | 24 (6.7) | 0.16 |
| Number of Attempts |  |  | 0.12 |
| 1 | 546 (86.9) | 289 (81.2) |  |
| 2 | 46 (7.3) | 38 (10.7) |  |
| 3 | 31 (4.9) | 17 (4.8) |  |
| 4 | 2 (0.3) | 6 (1.7) |  |
| 5 | 2 (0.3) | 1 (0.3) |  |
| Unknown | 1 (0.2) | 5 (1.4) |  |
| Current infection (yes) | 175 (27.9) | 106 (29.8) | 0.65 |
| Comorbidities |  |  | 0.30 |
| 1 | 93 (14.8) | 74 (20.8) |  |
| 2 | 89 (14.2) | 57 (16.0) |  |
| 3 | 50 (8.0) | 25 (7.0) |  |
| >3 | 322 (51.3) | 166 (46.6) |  |
| None | 74 (11.8) | 34 (9.6) |  |
| Side of Placement |  |  | 0.16 |
| Left | 409 (65.1) | 234 (65.7) |  |
| Right | 219 (34.9) | 119 (33.4) |  |
| Unknown | 0 (0.0) | 3 (0.8) |  |
| Number of lumens |  |  | 0.19 |
| 1 | 8 (1.3) | 7 (1.9) |  |
| 2 | 620 (98.7) | 346 (97.2) |  |
| Unknown | 0 (0.0) | 3 (0.8) |  |
| Ease of insertion^ǂ^ (mean, SD) | 87.1, 22.1 | 83.8, 23.2 | 0.03 |
| Inserter |  |  | 0.41 |
| Radiographer | 492 (78.3) | 279 (77.8) |  |
| Nurse | 132 (21.0) | 73 (20.5) |  |
| Doctor | 3 (0.5) | 2 (0.6) |  |
| Other | 1 (0.2) | 0 (0.0) |  |
| Unknown | 0 (0.0) | 4 (1.1) |  |
| **INTERVENTION/CONTROL** |  |  |  |
| ***PICNIC***^¶^ | ***n=568*** | ***n=349*** | 0.89 |
| Polyurethane PICC | 189 (33.2) | 119 (34.3) |  |
| Hydrophobic PICC | 190 (33.5) | 112 (32.3) |  |
| Chlorhexidine PICC | 189 (33.3) | 116 (33.4) |  |
| ***PISCES***^¶^ | ***n=60*** | ***n=9*** | 0.25 |
| ISD (+Chlorhexidine impregnated patch) | 19 (31.7) | 1 (11.1) |  |
| ISD (no Chlorhexidine impregnated patch) | 15 (25.0) | 5 (55.6) |  |
| SSD (+Chlorhexidine impregnated patch) | 13 (21.7) | 1 (11.1) |  |
| SSD (no Chlorhexidine impregnated patch) | 13 (21.7) | 2 (22.2) |  |
| **DEVICE CHARACTERISTICS** | **n (%)*** | **n (%)*** |  |
| **OUTCOMES** |  |  |  |
| Dwell time (in days)^#^ (mean, SD) | 29.6, 18.8 | 28.0, 18.6 | 0.21 |
| All-cause PICC complication | 79 (22.1) | 151 (24.0) | 0.59 |
| Complications^: |  |  |  |
| CLABSI | 12 (1.9) | 8 (2.3) | 0.92 |
| Local | 1 (0.3) | 3 (0.5) | 0.89 |
| Occlusion | 57 (9.1) | 19 (5.3) | 0.10 |
| Dislodgement | 21 (3.3) | 9 (2.5) | 0.75 |
| Confirmed Thrombosis | 21 (3.3) | 21 (5.9) | 0.16 |
| Fracture | 1 (0.2) | 3 (0.8) | 0.27 |
| Skin Reaction (yes)^¥^ | 13 (21.7) | 1 (11.1) | 0.001 |
| **RESPONSES** |  |  |  |
| **EQ5D-5L** |  |  |  |
| Utility at T0 (mean, SD) | 0.75, 0.28 | 0.75, 0.29 | 0.97 |
| Mobility |  |  | 0.10 |
| No | 348 (55.4) | 212 (59.6) |  |
| Slight | 124 (19.8) | 71 (19.9) |  |
| Moderate | 80 (12.7) | 27 (7.6) |  |
| Severe | 28 (4.5) | 22 (6.2) |  |
| Extreme | 48 (7.6) | 24 (6.7) |  |
| Personal care |  |  | 0.44 |
| No | 363 (57.8) | 216 (60.7) |  |
| Slight | 127 (20.2) | 73 (20.5) |  |
| Moderate | 81 (12.9) | 32 (9.0) |  |
| Severe | 31 (4.9) | 21 (5.9) |  |
| Extreme | 26 (4.1) | 14 (3.9) |  |
| Usual activities |  |  | 0.007 |
| No | 157 (25.0) | 124 (34.8) |  |
| Slight | 151 (24.0) | 60 (16.9) |  |
| Moderate | 167 (26.6) | 88 (24.7) |  |
| Severe | 53 (8.4) | 33 (9.3) |  |
| Extreme | 100 (15.9) | 51 (14.3) |  |
| Pain/discomfort |  |  | 0.57 |
| No | 198 (31.5) | 111 (31.2) |  |
| Slight | 210 (33.4) | 105 (29.5) |  |
| Moderate | 158 (25.2) | 106 (29.8) |  |
| Severe | 58 (9.2) | 32 (9.0) |  |
| Extreme | 4 (0.6) | 2 (0.6) |  |
| Anxiety/depression |  |  | 0.21 |
| No | 246 (39.2) | 130 (36.5) |  |
| Slight | 269 (42.8) | 143 (40.2) |  |
| Moderate | 98 (15.6) | 68 (19.1) |  |
| Severe | 11 (1.8) | 13 (3.7) |  |
| Extreme | 4 (0.6) | 2 (0.6) |  |

*Unless otherwise noted; ^†^comparisons using Chi-Square test (*Χ*²) for categorical variables and *t-*test for continuous variables; ^more than one category possible; ^ǂ^scale 0 to 100, higher score indicates greater ease, number of observations 566 & 341 for T1 responders and T1 non-responders, respectively; ^¶^study group/arm to which patient allocated; ^#^from insertion to removal or censoring at 8 weeks; ^¥^number of observations 60 and 9 for T1 responders and T1 non-responders, respectively; EQ5D-5L = EuroQual Five Dimension, Five Level; T0 = baseline timepoint; T1 = follow-up timepoint; CLABSI = central line-associated bloodstream infection; ISD = integrated securement dressing; PICC = peripherally inserted central catheter; SD = standard deviation
